# Supplementary material for: Synergistically optimized electron and phonon transport in high-performance copper sulfides thermoelectric materials via one-pot modulation
Source: Nat Commun. 2024 Mar 28;15:2736. doi: 10.1038/s41467-024-47148-0 (PMC10979026; doi:10.1038/s41467-024-47148-0)
Supplement: Supplementary file 1 — Supplementary Information [file 41467_2024_47148_MOESM1_ESM.pdf]

Supplementary materials

for

Synergistically optimized electron and phonon transport in high-performance copper sulfides thermoelectric materials via one-pot modulation

Yi-Xin Zhang[1], Qin-Yuan Huang[1], Xi Yan[1], Chong-Yu Wang[1], Tian-Yu Yang[1], Zi-Yuan Wang[1], Yong-Cai Shi[1], Quan Shan[1], Jing Feng[1] and Zhen-Hua Ge[1]*

[1]Faculty of Materials Science and Engineering, Kunming University of Science and Technology, Kunming 650093, China

*Correspondence to zge@kust.edu.cn (Z.G.)

This PDF file includes the following sections:

Supplementary Fig. 1 to Supplementary Fig. 17

Supplementary Table 1 to Supplementary Table 4

Calculation details

References

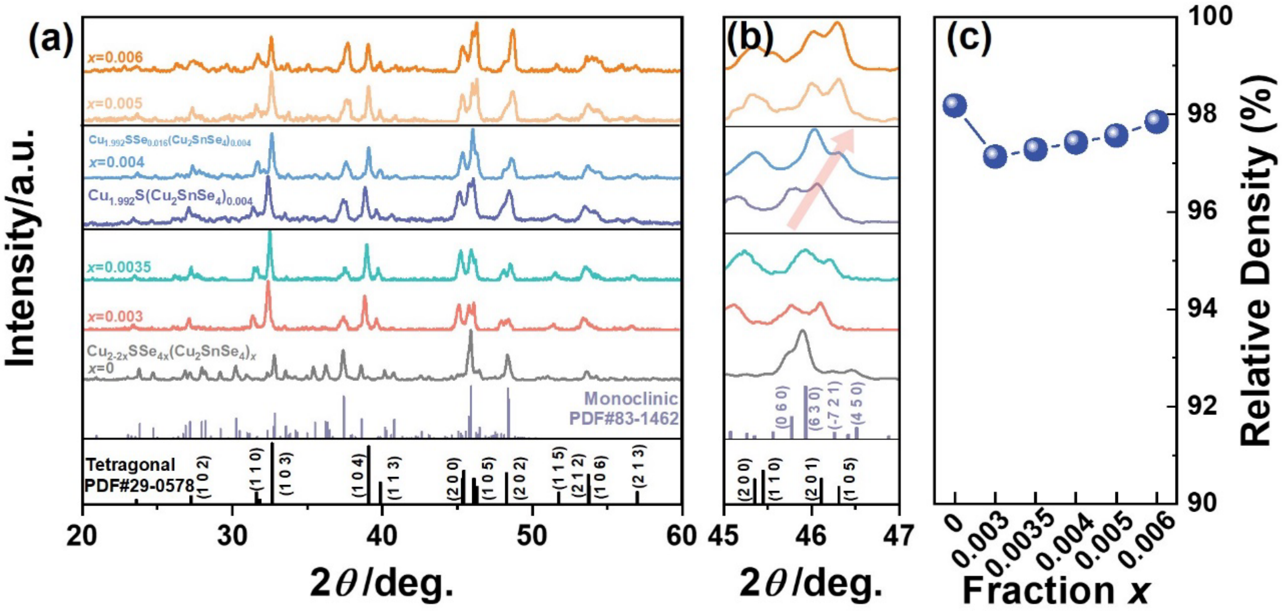

Supplementary Fig. 1 Phase structure of the copper sulfide-based bulk composites. (a) XRD patterns of the $Cu_{2-2x}SSe_{4x}(Cu_2SnSe_4)_x$ (x=0, 0.003, 0.0035, 0.004, 0.005 and 0.006) bulk specimens. (b) Enlarged patterns in the angle range of 45-47 degrees. (c) Relative density of all bulk composites.

As shown in Supplementary Fig. 2, nanopores are introduced in the copper sulfides after adding Sn and Se, which is mainly caused by the sulfur volatilization of the materials during the SPS process. Previous studies have proved that the pores within the grains are usually introduced by facilitating the S volatilization, additional nanoprecipitates prefer to distribute into the nanopores. Herein, the structure evolution of the copper sulfides results from the compositional regulation. There are tiny content of larger pores along the grain boundaries, which might be ascribed to the different thermal expansion between Cu_2S matrix and precipitates.

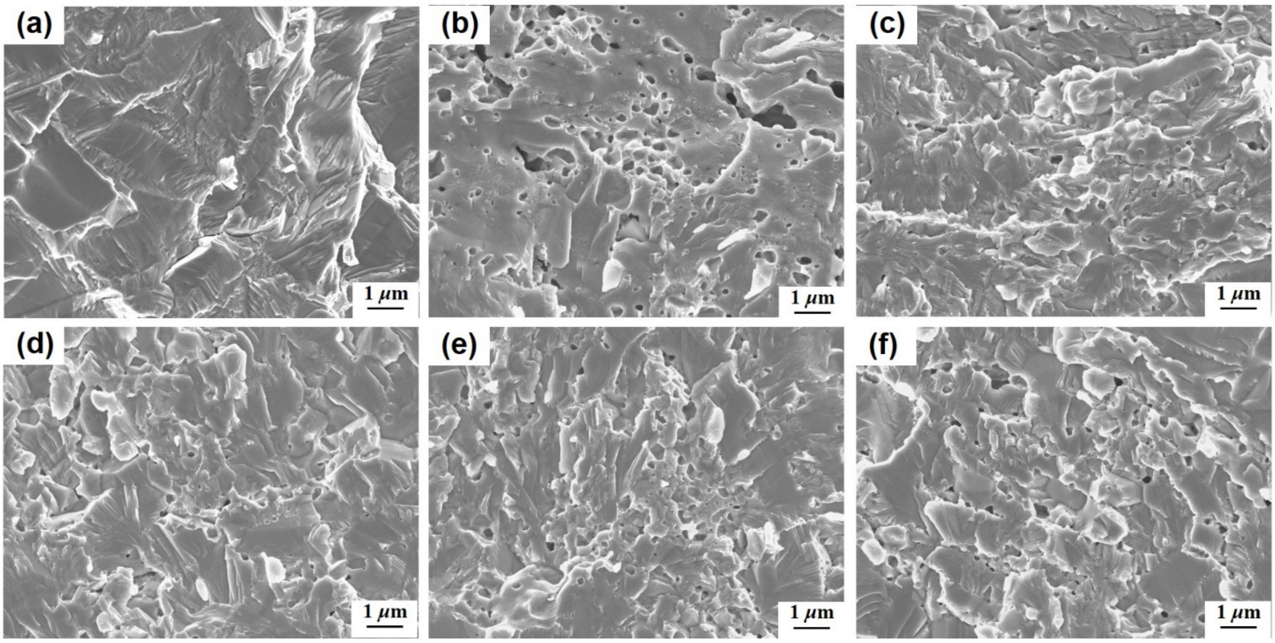

Supplementary Fig. 2 Morphology of the fractured $Cu_{2-2x}SSe_{4x}(Cu_2SnSe_4)_x$ (x=0, 0.003, 0.0035, 0.004, 0.005 and 0.006) bulk specimens. (a) x=0, (b) x=0.003, (c) x=0.0035, (d) x=0.004, (e) x=0.005, (f) x=0.006.

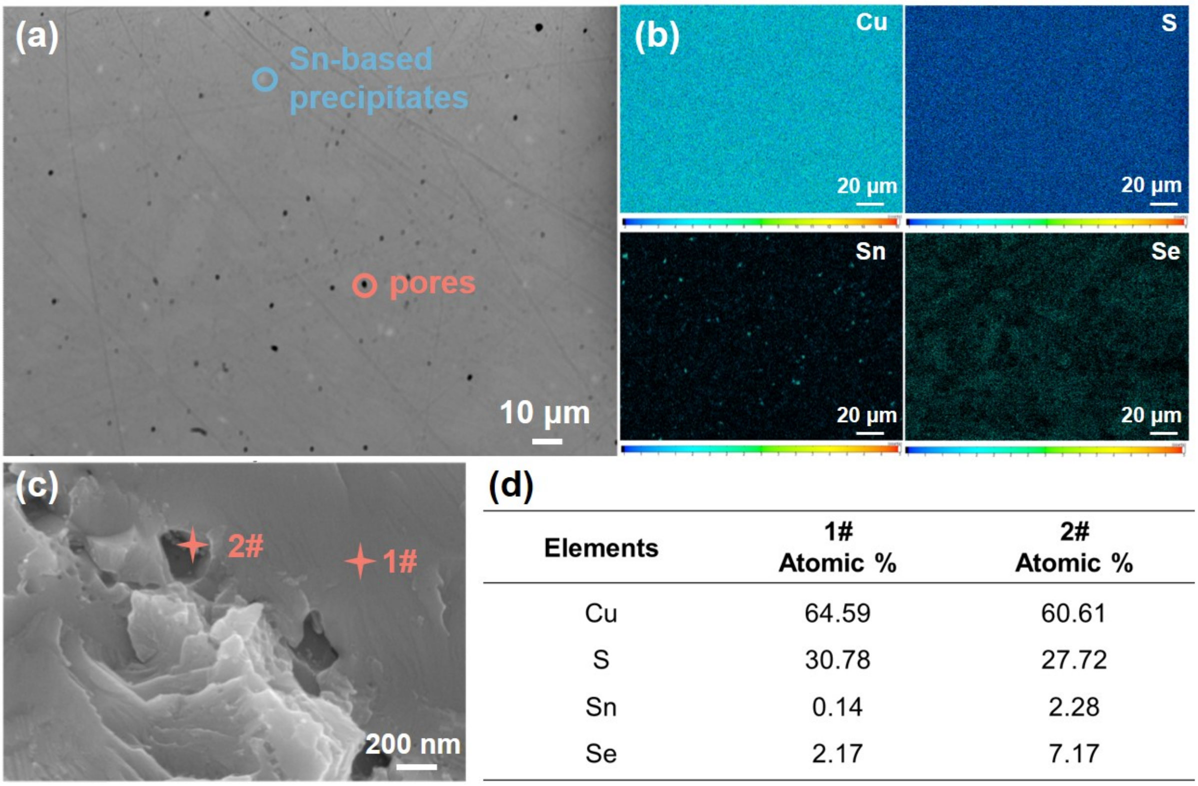

Elements	1# Atomic %	2# Atomic %
Cu	64.59	60.61
S	30.78	27.72
Sn	0.14	2.28
Se	2.17	7.17

Supplementary Fig. 3 Composition characterization of copper sulfide bulk by EPMA and EDS. (a) Backscattered electron (BSE) image of the $Cu_{1.992}SSe_{0.016}(Cu_2SnSe_4)_{0.004}$ sample. (b) The electron probe micro analyzer (EPMA) mapping for the polished specimen. (c) Morphology of the fractured $Cu_{1.992}SSe_{0.016}(Cu_2SnSe_4)_{0.004}$ sample. (d) Element content of the 1# and 2# position in part (c) by EDS spot scanning.

In order to characterize the effect of additional Se on the compositional evolution of the material, EPMA analyses were performed for the $Cu_{1.992}S(Cu_2SnSe_4)_{0.004}$ and $Cu_{1.992}SSe_{0.016}(Cu_2SnSe_4)_{0.004}$ samples, respectively. As shown in Supplementary Fig. 4, the ratio of Cu and S/Se in the $Cu_{1.992}S(Cu_2SnSe_4)_{0.004}$ sample is close to the pristine Cu_2S, indicating that the introduction of the designed content of Sn and Se in the Cu_2S would not obviously change the stoichiometric ratio of main phase. Furthermore, the introduction of extra Se without changing other condition can significantly decrease the Cu content of the matrix. Therefore, the ratio of Cu and S/Se for the $Cu_{1.992}SSe_{0.016}(Cu_2SnSe_4)_{0.004}$ sample reduces to about 1.96.

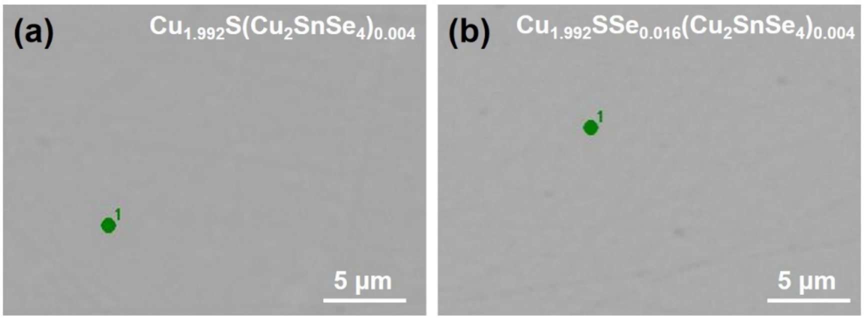

(c)

Elements	$Cu_{1.992}S(Cu_2SnSe_4)_{0.004}$	$Cu_{1.992}SSe_{0.016}(Cu_2SnSe_4)_{0.004}$
Cu (mol%)	66.18	66.12
S (mol%)	31.60	31.67
Se (mol%)	1.51	2.02
Cu/(S, Se)	2.00	1.96

Supplementary Fig. 4 EPMA results of the typical samples. Backscattered electron (BSE) images of the (a) $Cu_{1.992}S(Cu_2SnSe_4)_{0.004}$ and (b) $Cu_{1.992}SSe_{0.016}(Cu_2SnSe_4)_{0.004}$ sample, (c) the content of various elements in $Cu_{1.992}SSe_{0.016}(Cu_2SnSe_4)_{0.004}$ and $Cu_{1.992}S(Cu_2SnSe_4)_{0.004}$ specimens by EPMA.

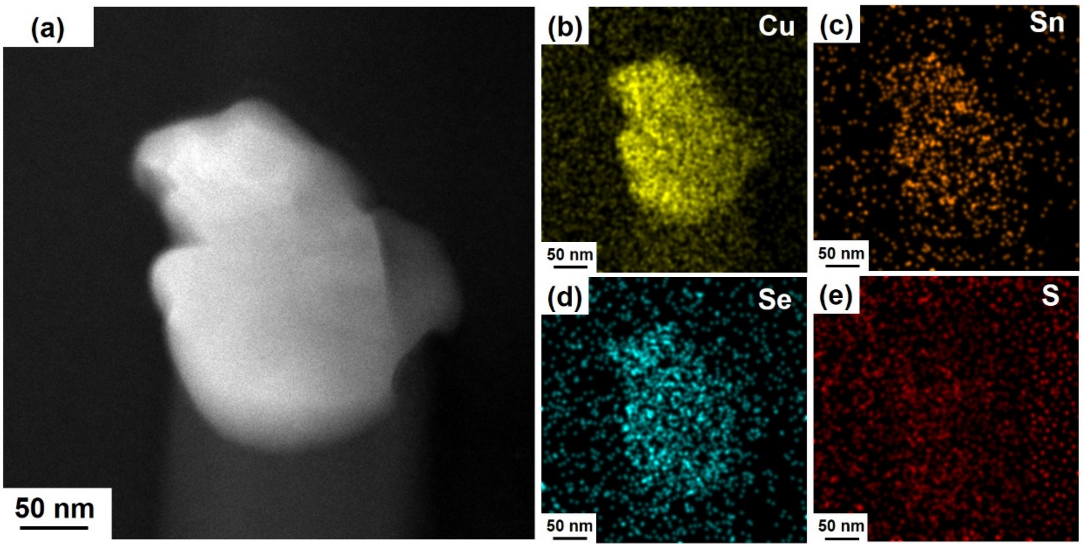

Supplementary Fig. 5 TEM characterization for the typical precipitate. (a) HAADF image of $Cu_{1.992}SSe_{0.016}(Cu_2SnSe_4)_{0.004}$ sample, showing the existence of nanoprecipitates. EDS mapping of the area in (a), (b) is Cu, (c) is Sn, (d) is Se and (e) is S.

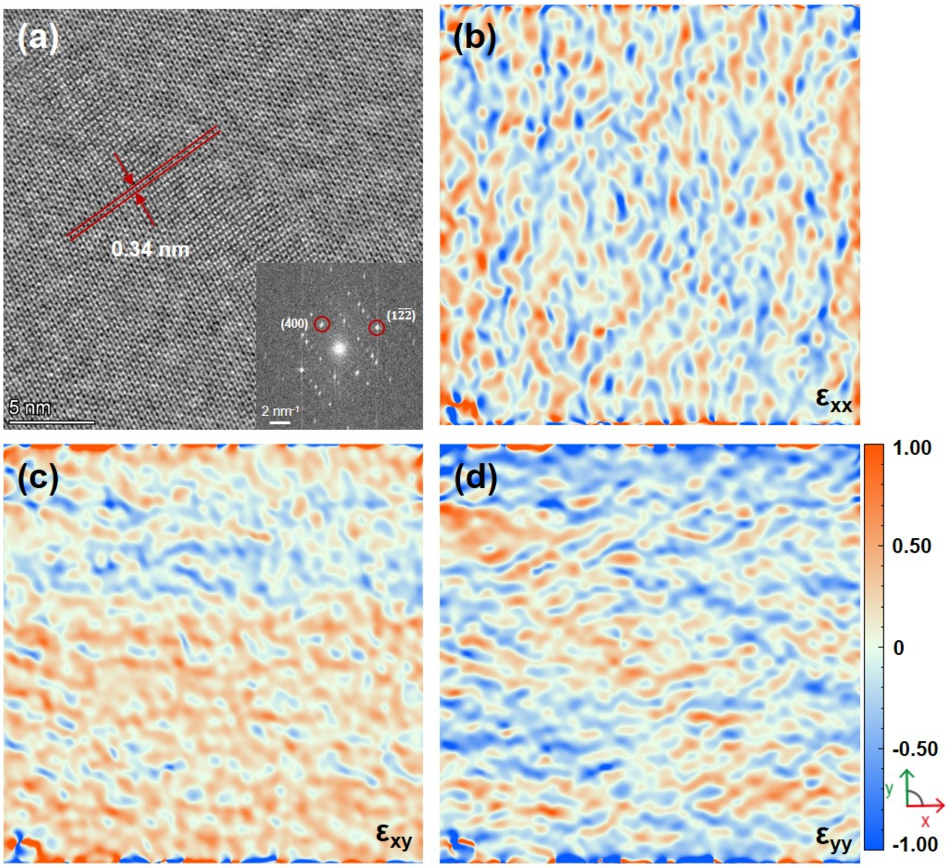

Supplementary Fig. 6 GPA of the pristine Cu_2S material. (a) HRTEM of the pristine Cu_2S material, the inset is the corresponding fast Fourier transform (FFT) image, (b-d) stress distribution of the pure Cu_2S material by geometric phase analysis.

There are two exothermic peaks at about 377 K and 720 K for the pure Cu_2S sample, corresponding to the monoclinic-hexagonal transition (Cu_2S M to H) and the hexagonal-cubic transition (Cu_2S H to C), respectively. Extra Sn and Se shifts these two phase transitions to the lower temperature due to the introduction of Cu vacancies. Additionally, an extra exothermic peak appears at about 418 K, which is consistent with the tetragonal-hexagonal transition. Although alloying with large content of Se in copper sulfides can remove the phase transition from the tetragonal phase (T) to the hexagonal phase, the adding content of Se in this study is tiny, and this phenomenon is not obvious. Therefore, adding Sn and Se can shift the two phase transitions (Cu_2S M to H and Cu_2S H to C) of copper sulfides to the lower temperature and introduce an extra phase transition ($Cu_{1.96}S$ T to H).

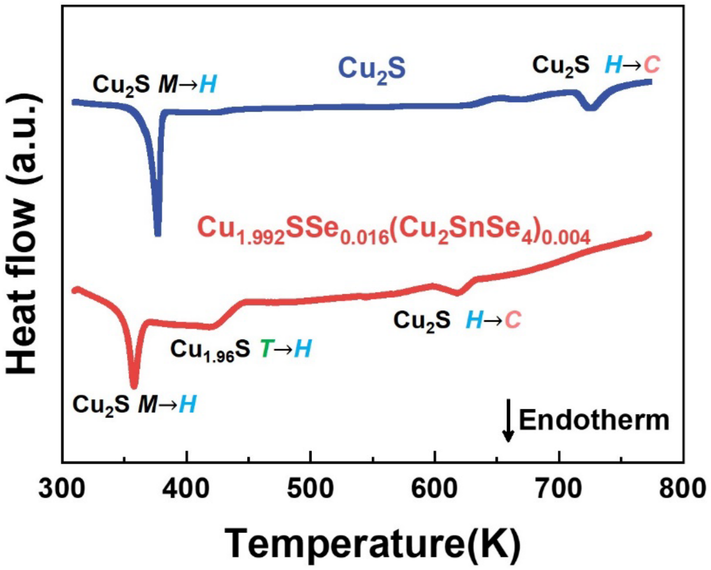

Supplementary Fig. 7 Differential scanning calorimetry (DSC) analysis of Cu_2S and $Cu_{1.992}SSe_{0.016}(Cu_2SnSe_4)_{0.004}$ samples in the temperature range of 310-773 K.

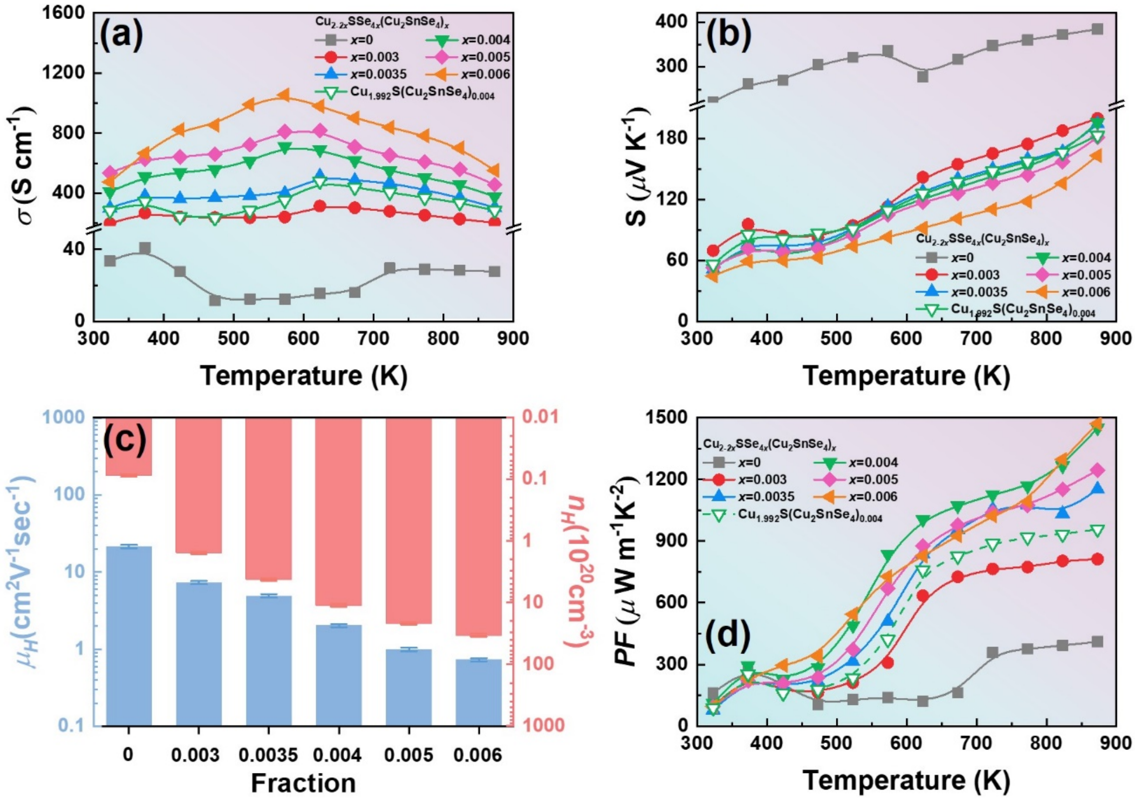

Supplementary Fig. 8 Electrical transport properties of all copper sulfide-based bulk composites. Temperature dependence of (a) electrical conductivity, b) Seebeck coefficient and (d) power factor of $Cu_{2-2x}SSe_{4x}(Cu_2SnSe_4)_x$ (x=0, 0.003, 0.0035, 0.004, 0.005 and 0.006) and $Cu_{1.992}S(Cu_2SnSe_4)_{0.004}$ bulk specimens. (c) Composition dependent hall carrier concentration and mobility at 300 K for all bulk samples. (d) $(\alpha h\upsilon)^2$ vs $h\upsilon$ of all specimens.

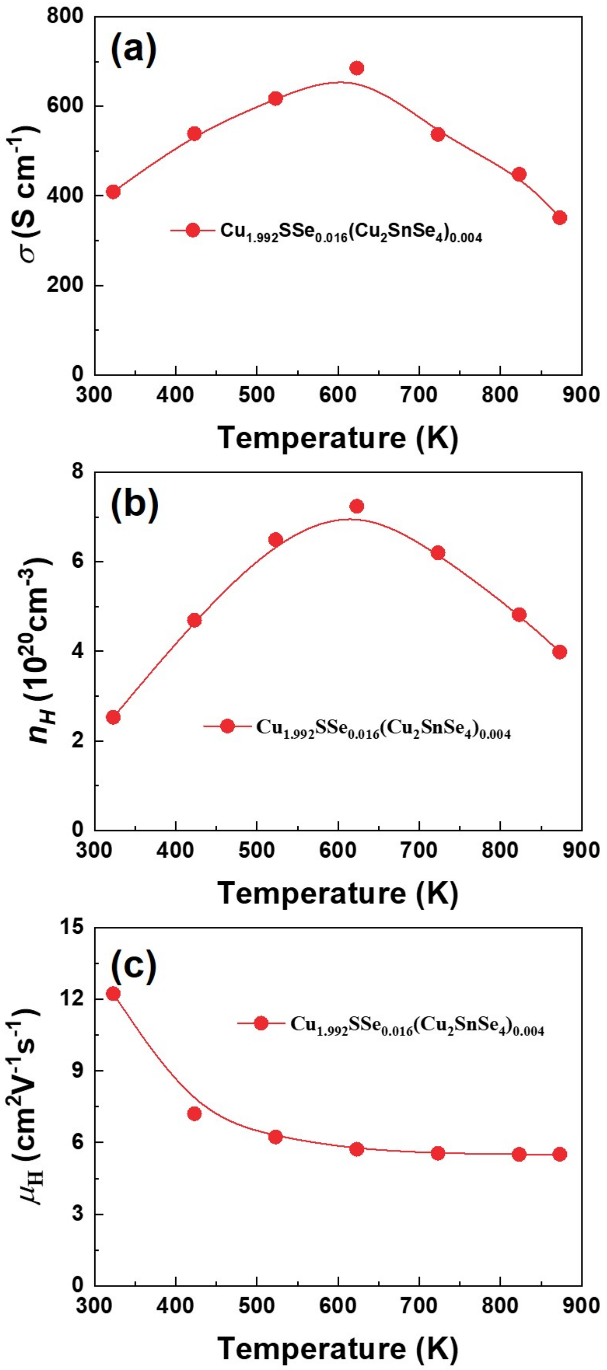

Supplementary Fig. 9 Hall measurement for $Cu_{1.992}SSe_{0.016}(Cu_2SnSe_4)_{0.004}$ bulk specimen at 300 K to 873 K. Temperature dependence of (a) electrical conductivity, (b) carrier concentration, and (c) carrier mobility of the $Cu_{1.992}SSe_{0.016}(Cu_2SnSe_4)_{0.004}$ bulk composites.

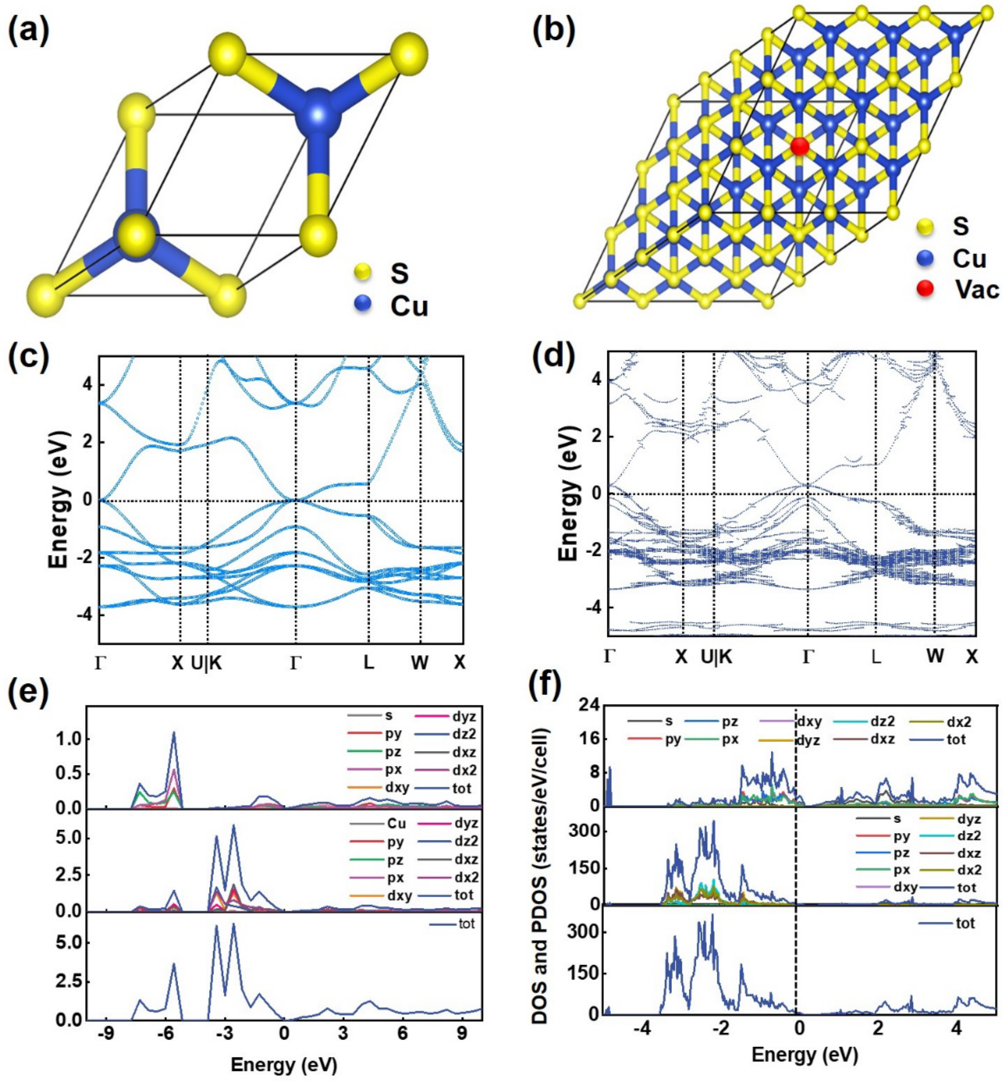

Supplementary Fig. 10 Electronic structural calculation results of Cu_2S and $Cu_{1.96}S$. (a) The unit cell structure of Cu_2S. (b) The supercells structure of $Cu_{1.96}S$ structure. (c) Effective band structure of Cu_2S supercells. (d) Effective band structure of $Cu_{1.96}S$ supercells. (e) TDOS and PDOS of Cu_2S supercells. (f) TDOS and PDOS of $Cu_{1.96}S$ supercells.

The density functional theory calculations were performed using the Vienna Ab initio Simulation Package (VASP.5.4.4)[1, 2]. The projected augmented wave approach (PAW)[3] and the exchange-correlation term of the generalized gradient approximation (GGA)[4] in the Perdew-Burke-Ernzerhof scheme (PBE)[5] were adopted. The 3×3×8 Monkhorst-Pack meshe of the k-point[6] was set to sample the first Brillouin zone, the planewave energy cutoff was 500 eV, and the VASP PREC flag was accurate. In addition, the energy convergence was set to 10-6 eV, and the force convergence was 0.001 eV/Å. It is worth noting that the $Cu_{1.96}S$ models were constructed using 3×3×4 supercells with 71 Cu atoms and 36 S atoms. Based on the optimized crystal structure, the electronic structure was then calculated. The charge density difference of $Cu_{1.96}S$ is displayed in Fig. 1; yellow indicates electron gain, and cyan-blue indicates electron loss. The linear Dirac dispersion near the Γ point suggests a high charge carrier mobility[7], which agrees well with the experimental results of high carrier mobility and good thermoelectric performance. In addition, Jonathan et al.[8] state that Cu vacancies could improve the mean squared displacement of Cu_2S, benefitting from increasing the ion diffusion rate in comparison with the Cu_2S material.

The electrical transport properties can be fitted by the single parabolic band (SPB) model, which assume a single, parabolic, and rigid band. The details of SPB model can be found everywhere. Using this model, the Seebeck coefficient (S), carrier concentration (n), carrier mobility (μ) can be calculated by the basic parameter (reduced chemical potential η, the mobility parameter m_0 and density of states effective mass m^*). These transport quantities are calculated by the following equations:

$$S = \frac{k_B}{e}\left(\frac{2F_1(\eta)}{F_0(\eta)} - \eta\right) \tag{S1}$$

$$\mathrm{n} = 4\pi\left(\frac{2m^* k_B T}{h^2}\right)^{1.5} F_{0.5}(\eta) \tag{S2}$$

$$F_i(\eta) = \int_0^\infty \frac{x^i\,dx}{1+exp(x-\eta)} \tag{S3}$$

$$n_H = \frac{n}{r_H} \tag{S4}$$

$$r_H = \frac{1.5F_{0.5}(\eta)(0.5)F_{-0.5}(\eta)}{F_0^2(\eta)} \tag{S5}$$

$$\mu_H = \mu_0 \frac{F_{-0.5}(\eta)}{2F_0(\eta)} \tag{S6}$$

$$\sigma = \frac{\mu_H}{R_H} \tag{S7}$$

Here, k_B is the Boltzmann's constant, $F_j(\eta)$ is the Fermi integral of order i, and the reduced chemical potential η is given by $\eta = E_F/(k_BT)$, where E_F is the Fermi energy.

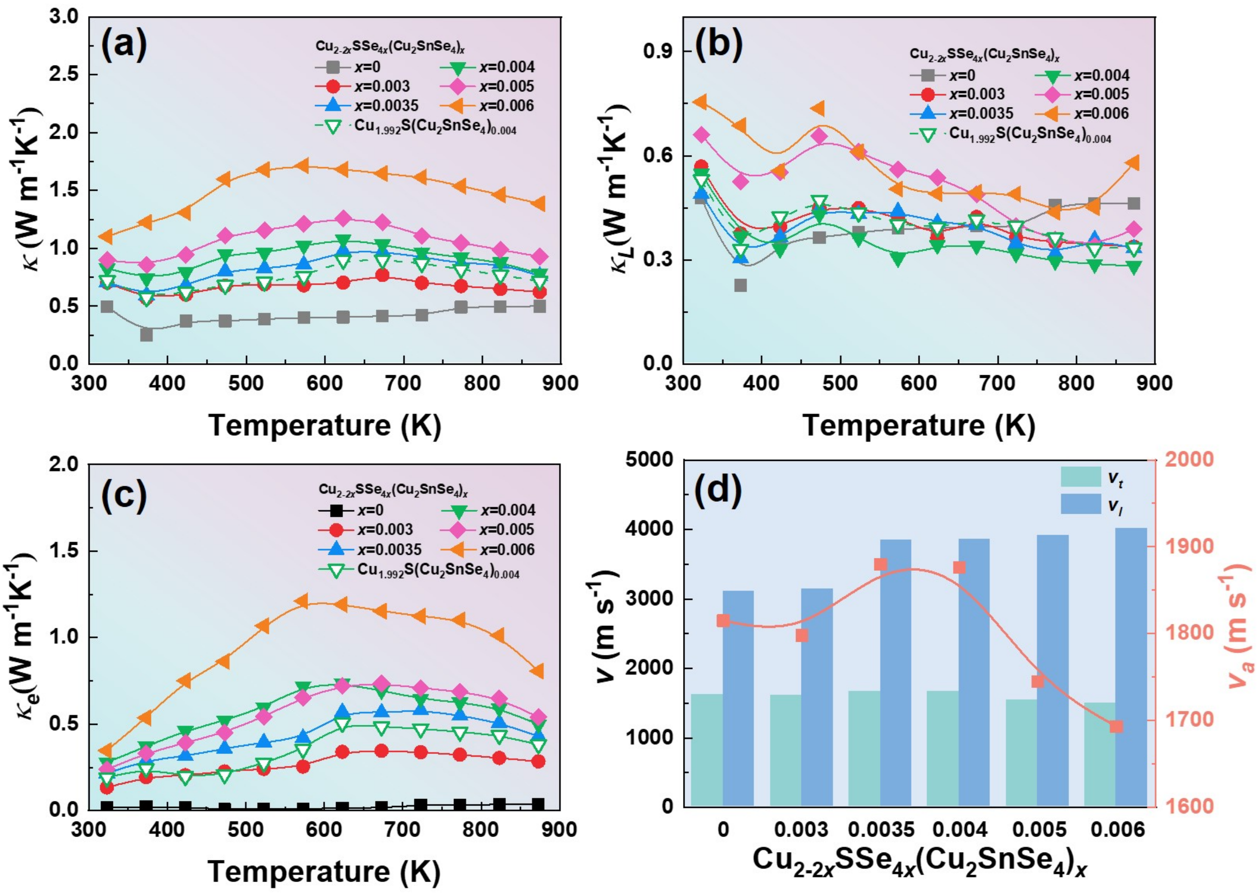

Supplementary Fig. 11 Thermal transport properties of all bulk composites. Temperature dependence of (a) total thermal conductivity, (b) lattice thermal conductivity, (c) carrier thermal conductivity, (d) composition dependence of sound velocity of $Cu_{2-2x}SSe_{4x}(Cu_2SnSe_4)_x$ (x=0, 0.003, 0.0035, 0.004, 0.005 and 0.006) and $Cu_{1.992}S(Cu_2SnSe_4)_{0.004}$ bulk specimens.

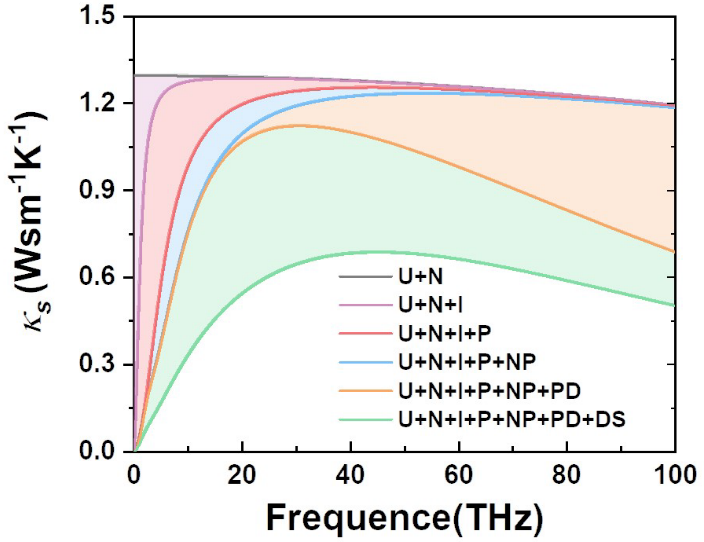

Supplementary Fig. 12 Spectral lattice thermal conductivity of the $Cu_{1.992}SSe_{0.016}(Cu_2SnSe_4)_{0.004}$ sample at 300 K. U (Umklapp), N (Normal), I (interfaces), P (precipitates), NP (nanoprecipitates), PD (point defect), and DS (grain boundary dislocation strain) are accounted for by the calculation.

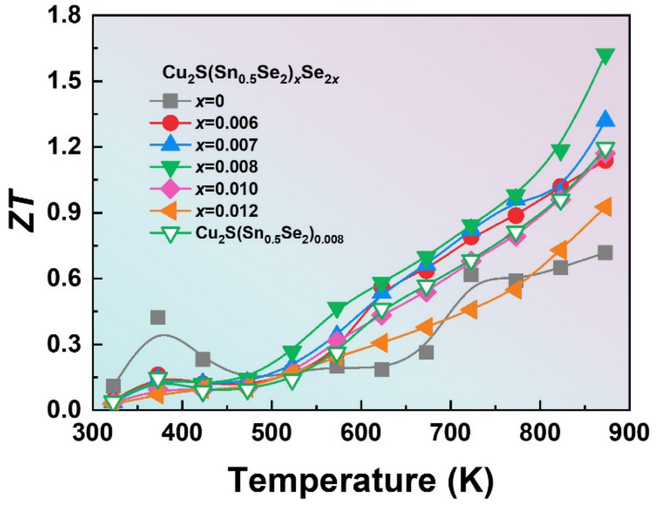

Supplementary Fig. 13 Thermoelectric figure of merit (*ZT*) for all bulk samples.

The maximum conversion efficiency (η_{max}) of heat to the electricity could be calculated by:

$$\eta_{max} = \eta_c \frac{\sqrt{1+(ZT)_{eng}(\bar{\alpha})/\eta_c - 1/2} - 1}{\alpha_0\sqrt{1+(ZT)_{eng}(\bar{\alpha})/\eta_c - 1/2} + 1 - \alpha_2} \quad \text{(S8)}$$

Where η_c is the Carnot efficiency that could be expressed as $(T_h\text{-}T_c)/T_h$, $(ZT)_{eng}$ is the engineering dimensionless figure of merit defined as

$$(ZT)_{eng} = \frac{(PF)_{eng}}{\int_{T_c}^{T_h} \kappa(T)dT} \Delta T \tag{S9}$$

$$(PF)_{eng} = \frac{(\int_{T_c}^{T_h} \alpha(T)dT)^2}{\int_{T_c}^{T_h} \rho(T)dT} \tag{S10}$$

$$\hat{\alpha} = \frac{\alpha(T_h)\Delta T}{\int_{T_c}^{T_h} \alpha(T)dT} \tag{S11}$$

$$\alpha_i = \frac{S(T_h)\Delta T}{\int_{T_c}^{T_h} \alpha(T)dT} - \frac{\int_{T_c}^{T_h} \tau(T)dT}{\int_{T_c}^{T_h} \alpha(T)dT} W_T \eta_c - iW_J \eta_c \tag{S12}$$

τ(T) is the temperature dependent Thomson coefficient defined as:

$$\tau(T) = T\frac{dS(T)}{dT} \tag{S13}$$

W_J and W_T are defined as dimensionless weight factors of Joule and Thomson heat, respectively, which could be expressed by:

$$W_J = \frac{\int_{T_c}^{T_h} \int_{T}^{T_h} \rho(T)dTdT}{\Delta T \int_{T_c}^{T_h} \rho(T)dT} \tag{S14}$$

$$W_T = \frac{\int_{T_c}^{T_h} \int_{T}^{T_h} \tau(T)dTdT}{\Delta T \int_{T_c}^{T_h} \tau(T)dT} \tag{S15}$$

$(PF)_{\mathrm{eng}}$ is the engineering power factor, $\hat{\alpha}$ defines the Thomson effect intensity, $\alpha(T_h)$ is the Seebeck coefficient at hot side.

The output power density at the maximum efficiency could be expressed as:

$$P_d = \frac{(PF)_{eng}\Delta T}{L} \frac{m_{opt}}{(1+m_{opt})^2} \tag{S16}$$

P_{d} is dependent to the TE leg's dimensions (L) and the intrinsic properties of TE materials. The optimized ratio m_{opt} could be obtained by:

$$m_{opt} = \sqrt{1 + (ZT)_{eng}\left(\frac{\hat{\alpha}}{\eta_c} - \frac{1}{2}\right)} \tag{S17}$$

In this study, the T_h is 750 K, the T_c is 300 K, the L is set as 2 mm.

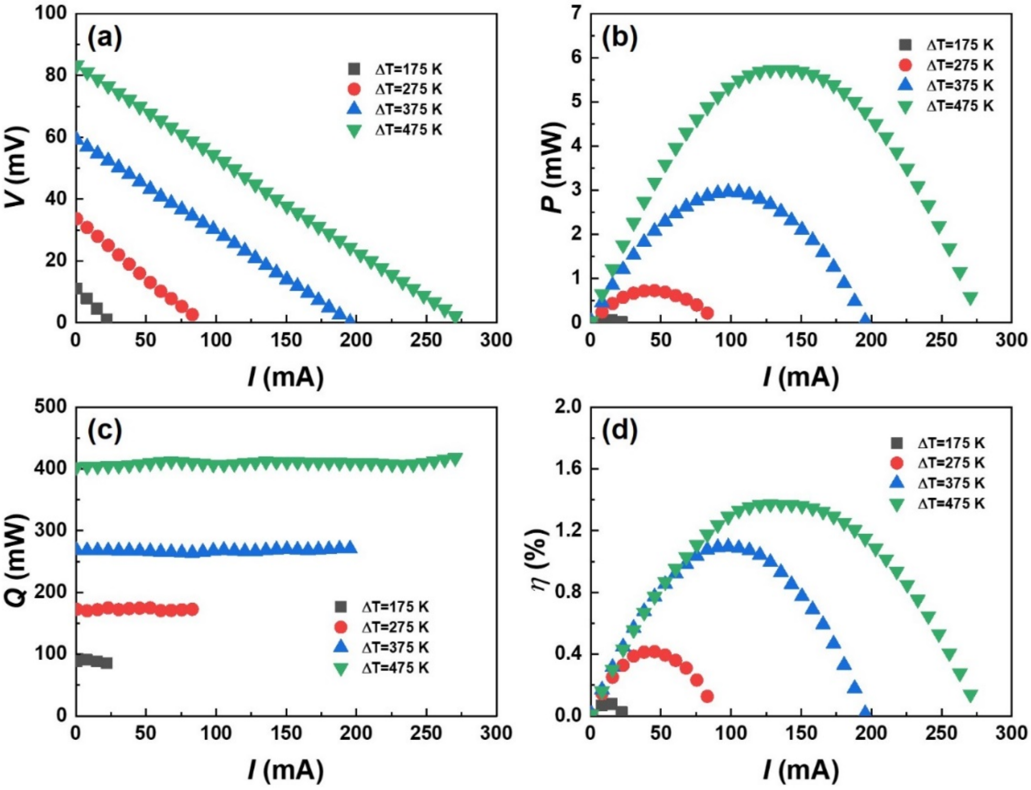

Supplementary Fig. 14 Thermoelectric conversion efficiency measured by mini-PEM instrument. Current-dependent (a) voltage, (b) output power, (c) heat flux, and (d) conversion efficiency at different hot-side temperatures for the single-leg thermoelectric module made by $Cu_{1.992}SSe_{0.016}(Cu_2SnSe_4)_{0.004}$ material.

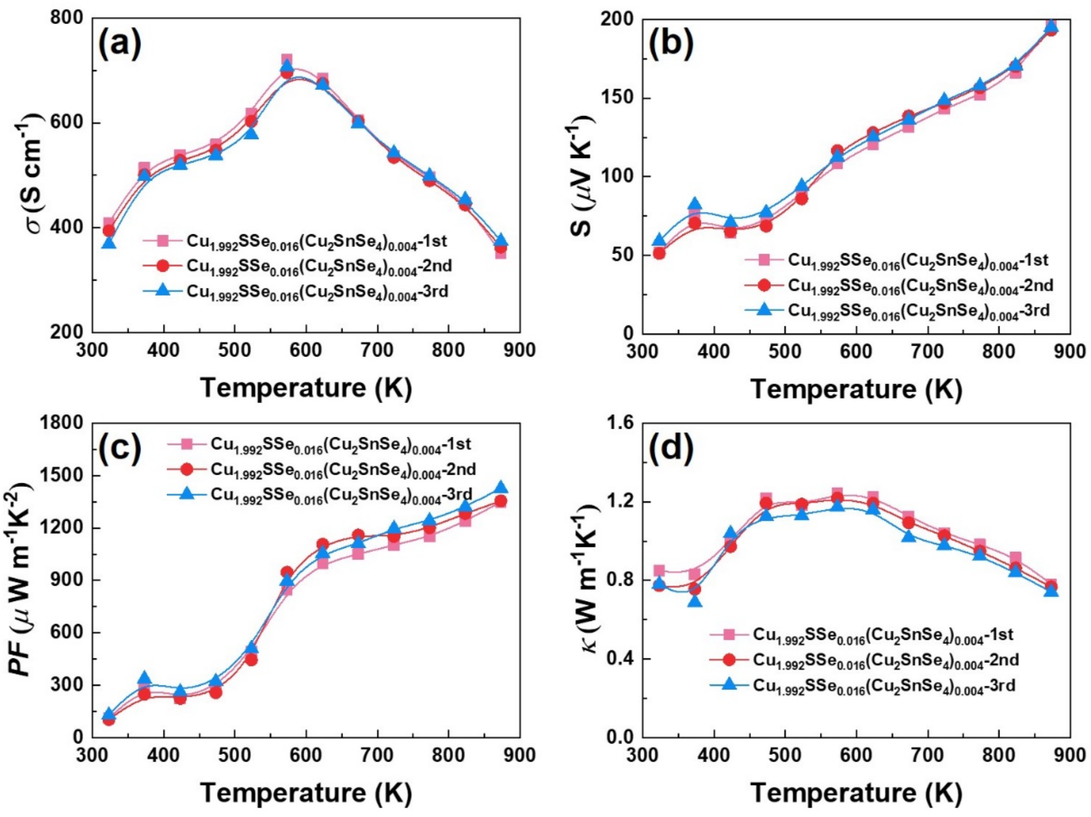

Supplementary Fig. 15 Cycling measurement of $Cu_{1.992}SSe_{0.016}(Cu_2SnSe_4)_{0.004}$ bulk specimens. The temperature dependence of (a) electrical conductivity, (b) Seebeck coefficient and (c) power factor and (d) thermal conductivity of the $Cu_{1.992}SSe_{0.016}(Cu_2SnSe_4)_{0.004}$ bulk specimens in the cycling measurement.

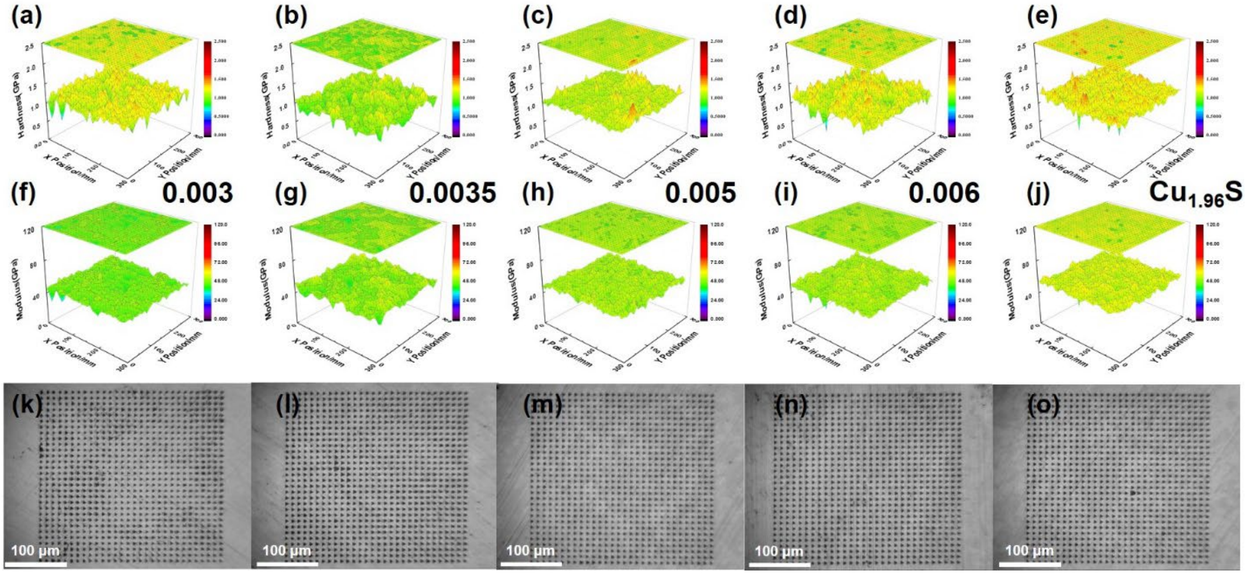

Supplementary Fig. 16 Mechanical performance of $Cu_{1.96}S$ and $Cu_{2-2x}SSe_{4x}(Cu_2SnSe_4)_x$ (x=0.003, 0.0035, 0.005 and 0.006) bulk specimen by Nanoindentation instrument. (a-e) Vickers hardness of the $Cu_{2-2x}SSe_{4x}(Cu_2SnSe_4)_x$ (x= 0.003, 0.0035, 0.005 and 0.006) and $Cu_{1.96}S$ samples, respectively. (f-j) Young's modulus of the $Cu_{2-2x}SSe_{4x}(Cu_2SnSe_4)_x$ (x= 0.003, 0.0035, 0.005 and 0.006) and $Cu_{1.96}S$ samples, respectively. (k-o) Corresponding nanoindentation images of the $Cu_{2-2x}SSe_{4x}(Cu_2SnSe_4)_x$ (x= 0.003, 0.0035, 0.005 and 0.006) and $Cu_{1.96}S$ samples.

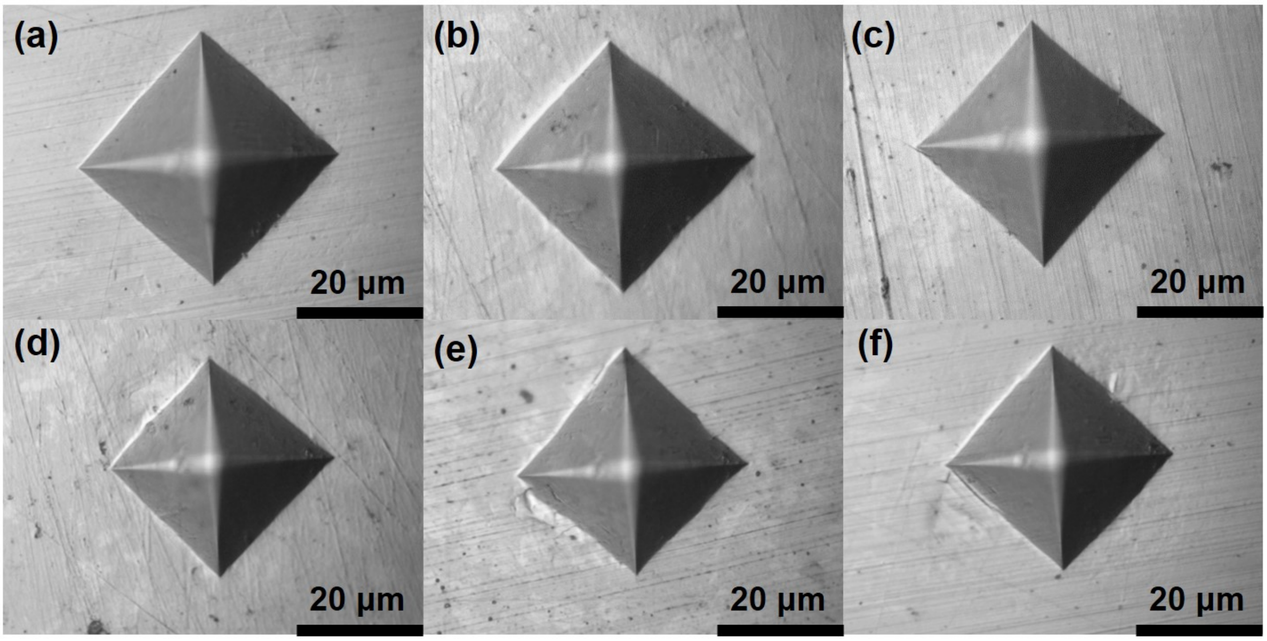

Supplementary Fig. 17 Nanoindentation images of the $Cu_{2-2x}SSe_{4x}(Cu_2SnSe_4)_x$ (x=0, 0.003, 0.0035, 0.004, 0.005 and 0.006) samples that were measured by microhardness tester, (a) is x=0, (b) is x=0.003, (c) is x=0.035, (d) is x=0.004, (e) is x=0.005 and (f) is x=0.006.

Average sound velocity (v_a) is calculated from the sound velocity as follows.

$$\frac{1}{v_a} = \left[\frac{1}{3}\left(\frac{1}{v_l^3} + \frac{2}{v_t^3}\right)\right]^{1/3} \quad \text{(S18)}$$

where v_l is the longitudinal sound velocity and v_t is the transverse sound velocity.

Young's modulus (E) is calculated by:

$$E = \frac{\rho v_t^2 (3v_l^2 - 4v_t^2)}{(v_l^2 - v_t^2)} \quad \text{(S19)}$$

where ρ is the sample density.

Poisson ratio (r) is calculated by[2]

$$r = \frac{1-2(v_t/v_l)^2}{2-2(v_t/v_l)^2} \quad \text{(S20)}$$

Shear modulus (G) is calculated by[2]

$$G = \frac{E}{2(1+r)} \quad \text{(S21)}$$

The Gruneisen parameter (γ) is calculated by[2]

$$\gamma = \frac{3}{2}\left(\frac{1+r}{2-3r}\right) \quad \text{(S22)}$$

Debye temperature (θ_D) is calculated by[2]

$$\theta_D = \frac{h}{k_B}\left(\frac{3N}{4\pi V}\right)^{1/3} v_a \quad \text{(S23)}$$

where h is Planck's constant, k_B is the Boltzmann constant, N is the number of atoms in the primitive unit cell (N = 8 for PbS) and V is the unit cell volume.

Bulk modulus (B) is calculated by[2]

$$B = \rho v_l^2 - \frac{4}{3}G \quad \text{(S24)}$$

Supplementary Table 1 Density (ρ), longitudinal (v_l), shear (v_t), average acoustic velocities (v_a), Young's modulus (E) and shear modulus (G) measured by ultrasonic reflection method at room temperature, Poisson's ratio (r), Grüneisen parameters (γ) of $Cu_{2-2x}SSe_{4x}(Cu_2SnSe_4)_x$ (x=0, 0.003, 0.0035, 0.004, 0.005 and 0.006) samples.

**Sample**	ρ (gcm^{-3})	v_l (ms^{-1})	v_t (ms^{-1})	v_a (ms^{-1})	E (GPa)	G (GPa)	r	γ
Cu_2S	5.57	3111	1621	1815	38.5	14.6	0.314	1.86
$Cu_{1.992}SSe_{0.012}(Cu_2SnSe_4)_{0.003}$	5.60	3145	1604	1798	38.2	14.4	0.324	1.93
$Cu_{1.992}SSe_{0.014}(Cu_2SnSe_4)_{0.0035}$	5.60	3844	1664	1880	42.9	15.5	0.385	2.455
$Cu_{1.992}SSe_{0.016}(Cu_2SnSe_4)_{0.004}$	5.61	3858	1660	1876	42.9	15.5	0.386	2.473
$Cu_{1.992}SSe_{0.020}(Cu_2SnSe_4)_{0.005}$	5.62	3913	1539	1745	37.5	13.31	0.409	2.728
$Cu_{1.992}SSe_{0.024}(Cu_2SnSe_4)_{0.006}$	5.68	4009	1491	1693	35.9	12.63	0.420	2.875
$Cu_{1.96}S$	5.69	3828	1209	1377	24.0	8.32	0.445	3.253

Phonon transport modeling

The Callaway model is expressed as follows,

$$\kappa_L = \frac{k_B}{2\pi^2 v_a}\left(\frac{k_B T}{\hbar}\right)^3 \int_0^{\theta_D/T} \frac{x^4 e^x}{\tau_C^{-1}(e^x-1)^2} dx \quad \text{(S25)}$$

where $x = \hbar\omega/k_B T$, ω, k_B, $\hbar$, θ_D and τ_C are reduced phonon frequency, phonon frequency,

Boltzmann constant, reduced Planck constant, Debye temperature and the overall phonon scattering relaxation time, respectively. The overall phonon scattering relaxation time is expressed as:

$$\tau_C^{-1} = \tau_I^{-1} + \tau_U^{-1} + \tau_{PD}^{-1} + \tau_{GBDS}^{-1} \tag{S26}$$

The relaxation time of phonons is independent with phonon frequencies. Thus, the frequency-independent τ_I is given by

$$\tau_I^{-1} = \frac{v_a}{L} \tag{S27}$$

Where v_a is the average sound velocity and L is the experimentally determined grain size.

Umklapp scattering is the dominant process in material, describing the interaction of phonons, whose relaxation time is of the form

$$\tau_U^{-1} = A_N \frac{2}{(6\pi^2)^{1/3}} \frac{k_B V^{1/3} \gamma^2 \omega^2 T}{M v_a^3} \tag{S28}$$

Where V, M are atomic volume and atomic mass, respectively. The parameter A_N is the ratio of Normal to Umklapp process. All the parameters used in the calculation are listed in Table S2. The alloyed Se atoms will also introduce mass and lattice constant fluctuations, resulting in point defect scattering for phonons. The relaxation time of point defect scattering can be written as

$$\tau_{PD}^{-1} = \frac{V\omega^4}{4\pi v_a^3}\Gamma \tag{S29}$$

Where Γ is the scattering parameter related to mass (ΔM) and lattice constant (Δa) differences between two constituents of an alloy. The result indicates that the point defect scattering is a second reason for the largely depressed lattice thermal conductivity.

The relaxation time of GBDS is written as two parts: cores τ_{DC} and strain τ_{DS},

$$\tau_{DC}^{-1} = N_d \frac{V^{4/3}}{v^2} \omega^3 \tag{S30}$$

$$\tau_{DS}^{-1} = 0.6 B_D^2 N_d (\gamma + \gamma_1)^2 \omega \left[\frac{1}{2} + \frac{1}{24}\left(\frac{1-2r}{1-r}\right)^2 \left\{1 + \sqrt{2}\left(\frac{v_l}{v_t}\right)^2\right\}^2\right] \tag{S31}$$

Where B_D, r, γ and γ_1 are the magnitude of effective Burgers vector, the Poisson ratio, Gruneisen

parameter and change in Gruneisen parameter, respectively.

Supplementary Table 2 Key parameters for Debye-Callaway modeling

Parameters	Notes	Values
v_a	Average sound velocity	1876 ms^{-1}
L	Average grain size	7.05 μm
A_N	Ratio of Normal to Umklapp process	2.5
V	Average atomic volume	7.38×10^{-30} m^3
M	Average atomic mass	8.81×10^{-26} kg
γ	Gruneisen parameter	2.47
$\boldsymbol{N_D}$	Density of GBDS	3×10^{16} cm^{-2}
$\boldsymbol{B_D}$	Effective Burgers vector	0.4 nm
γ_I	Change of Gruneisen parameter	0.05
R	Poisson ratio	0.386
θ_D	Debye Temperature	222.38

Supplementary References

1. Hohenberg P., Kohn W. Density functional theory (DFT). *Phys. Rev. B.* **136**, 864-871 (1964).

2. Kresse G., Furthmuller J. Efficient iterative schemes for ab initio total-energy calculations using a plane-wave basis set. *Phys. Rev. B.* **54**, 11169 (1996).

3. Blöchl P. E. Projector augmented-wave method. *Phys. Rev. B.* **50**, 17953 (1994).

4. Perdew J. P., Burke K., Ernzerhof M. Generalized gradient approximation made simple. *Phys. Rev. Lett.* **77**, 3865-3868 (1996).

5. Perdew J. P., Wang Y. Accurate and simple analytic representation of the electron-gas correlation energy. *Phys. Rev. B.* **45**, 13244-13249 (1992).

6. Monkhor H. J., Pack J.D. Special points for Brillouin-zone integrations. *Phys. Rev. B.* **13**, 5188-5192

(1976).

7. Kurosaki K., Kosuga A., Muta H., Uno M., Yamanaka S. Ag_9TlTe_5: A high-performance thermoelectric bulk material with extremely low thermal conductivity. *Appl. Phys. Lett.* **87**, 061919 (2005).
8. Jia T. T., Chen G., Zhang Y. S. Lattice thermal conductivity evaluated using elastic properties. *Phys. Rev. B* **95**, 155206 (2017).
9. Boukhris N., Meradji H., Korba S. A., Drablia S., Ghemid S., Hassan F. E. First principles calculations of structural, electronic and thermal properties of lead chalcogenides PbS, PbSe and PbTe compounds. *B Mater. Sci.* **37**, 1159-1166 (2014).
10. Cardona M., Kremer R. K., Lauck R., Siegle G., Serrano J., Romero A. H. Heat capacity of PbS: Isotope effects. *Phys. Rev. B* **76**, 075211 (2007).
11. Callaway J. Model for lattice thermal conductivity at low temperatures. *Phys. Rev.* **113**, 1046-1051 (1959).
12. Kim H. S., Kang S. D., Tang Y. L., Hanus R., Snyder G. J. Dislocation strain as the mechanism of phonon scattering at grain boundaries. *Mater. Horiz.* **3**, 234-240 (2016).
13. Wang H., Pei Y. Z., LaLonde A. D., Snyder G. J. Weak electron–phonon coupling contributing to high thermoelectric performance in n-type PbSe. *P. Natl. Acad. Sci. U.S.A.* **109**, 9705-9709 (2012).
14. Abeles B. Lattice thermal conductivity of disordered semiconductor alloys at high temperatures. *Phys. Rev.* **131**, 1906-1911 (1963).
15. Slack G. A. Effect of isotopes on low-temperature thermal conductivity. *Phys. Rev.* **105**, 829-831 (1957).
16. Zou J., Kotchetkov D., Balandin A. A., Florescu D. I., Pollak F. H. Thermal conductivity of GaN films: Effects of impurities and dislocations. *J. Appl. Phys.* **92**, 2534-2539 (2002).
